# Avoid Using Phosphate Buffered Saline (PBS) as an Electrolyte for Accurate OER Studies

**DOI:** 10.1021/acsenergylett.4c01589

**Published:** 2024-07-18

**Authors:** Anthony R. Kucernak, Haiyi Wang, Xiaoqian Lin

**Affiliations:** † Department of Chemistry, Imperial College London, London W12 0BZ, United Kingdom; ‡ Department of Chemistry, Imperial College London, White City, London W12 0BZ, United Kingdom

Water electrolysis has been attractive to store renewable electricity into hydrogen fuel with good efficiency and high gas purity.^1,2^ The hydrogen evolution reaction (HER) is usually coupled with the more sluggish oxygen evolution reaction (OER). Hence, efforts are focused on developing effective catalysts to lower the high OER overpotential, enhancing the overall electrolyzer performance.^3,4^ Precious metals like iridium oxide and ruthenium oxide serve as excellent OER electrocatalysts with high activity and stability in both acidic and alkaline media.^3,5^ Earth-abundant transition-metal based electrocatalysts such as Fe, Co, and Ni are popular alternatives to precious metals, but they show stability over a more limited pH range.^6−11^ Interest is also growing in developing OER electrocatalysts at near neutral pHs, alleviating safety concerns and minimizing capital costs associated with extreme pH environments.^11−14^

PBS (phosphate buffered saline), a pH 7.4 buffer solution, is widely employed in biological research for its nontoxicity to cells. The composition of “1×” PBS is shown in Table S2, with “saline” alluding to the major component, sodium chloride (NaCl). Recently, it has gained popularity as an electrolyte in assessing the performance of newly developed OER catalysts under neutral condition.^15−20^ A standard 1× PBS solution has a reasonably good ionic strength of 0.156 M, providing adequate ionic conductivity for electrochemical reactions. The presence of phosphate buffer provides a fast reactant supply (OH^–^) and reduces local pH shift during OER, better revealing the intrinsic catalytic properties. However, the low concentration of phosphate and the pH offset from the p*K*_a_ of the H_2_PO_4_^–^/ HPO_4_^2–^ couple mean that the pH will undergo a considerable shift at currents > 1 mA cm^–2^ on a rotating disk electrode (RDE) (and even more on a static system).^21^ Despite this, it is still desirable to keep the concentration of phosphate low, as a moderate adsorbing effect of phosphate onto the active sites of IrO_*x*_ under neutral conditions has been reported.^22^

The prevalent use of “0.1/1 M PBS” in the water electrolysis community often lacks composition details,^23−26^ with “PBS” sometimes being used without specifying its full name.^27−30^ This leads to misinformation within the water electrolysis community, as it could either denote pure phosphate buffer solution^31−33^ or phosphate buffered saline,^15−20^ which have different salt compositions, ionic strengths and ionic conductivities. Such lack of clarity poses complication and misinformation during cross-referencing among researchers.^23,24^ Furthermore, denoting concentration using molarity is only correct for pure phosphate buffer solution, but not for phosphate buffered saline. Denoting such composition of phosphate buffered saline using “1.0 M” instead of “1×” is incorrect, as neither the salt concentration nor the ionic strength of the solution is 1 M. Researchers may have inadvertently prepared 1× PBS with either 1 M phosphate salt or 1 M NaCl instead of the correct 139.7 mM NaCl/KCl composition. A precise composition description is essential to accurately convey the actual composition of PBS in the scientific literature.

Unlike researchers specializing in seawater electrolysis, those developing OER catalysts for water electrolysis under neutral conditions often overlook the chemistry of chloride-containing solutions. Chloride ions, present in 1× PBS at a concentration of 0.139 M, could be oxidized to hypochlorite concurrently with water oxidation. Thermodynamically, the hypochlorite formation reaction (HCFR, eqs 1 and 2) is less favorable compared to the OER (eq 4) under neutral conditions. However, the sluggish four-electron-transfer OER process, which requires a large overpotential, falls within the electrochemical potential of HCFR, a more kinetically facile two-electron-transfer reaction.
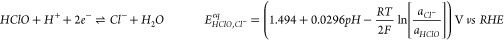
1
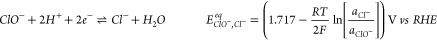
2
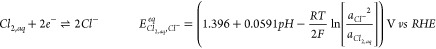
3
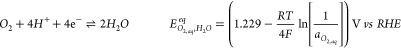
4

Equations 1–4 show possible electrochemical reactions calculated from thermodynamic data and corrected to the reversible hydrogen electrode (RHE) scale.^34^ The equations also show the dependence on the activities of the reactants and products. At PBS buffer’s pH (7.4), the HCFR equilibrium potentials are higher than the OER equilibrium potential (1.713 V, 1.717 V, 1.833 V, 1.223 V vs RHE at unit activity for eqs 1–4). The most thermodynamically facile chloride reactions are eqs 1 and 2, which show similar values, as the buffer pH is very close to the p*K*_a_ of HClO (7.56, calculated from thermodynamic data).^34^

Benchmark OER catalysts, including iridium- and ruthenium-oxide materials, demonstrate catalytic activity toward chloride oxidation under neutral conditions.^35,36^ For instance, IrO_2_/RuO_2_ coated TiO_2_ is often used as anode in the chlor-alkali process.^37^ Moreover, many earth-abundant transition metals, such as Co(OH)_2_,^38^ Fe_3_O_4_,^39^ and PbO_2_,^40^ show a preference for chloride oxidation over OER in chloride-containing neutral electrolytes.^41^ Chloride ions are also well-known in enhancing metal corrosion by forming stable, soluble metal-chloride complexes through strong coordination with metal cations, causing anodic current rise before OER.^42^ This corrosion issue has gained attention in seawater electrolysis but received minimal focus in water electrolysis.^6,7,43−45^ Consequently, claims of excellent OER activity for new catalysts in PBS without addressing these issues may be unreliable. HCFR and catalyst corrosion may significantly contribute to the observed current, rendering false interpretation of the catalyst’s performance and mechanism.

This study aims to evaluate the impact of chloride ions in PBS on the observed OER performance. PBP (phosphate-buffered perchlorate), which replaces Cl^–^ with ClO_4_^–^ at the same concentration, emerges as a better alternative to PBS due to the low absorptivity of ClO_4_^–^ at active catalyst sites. Notably, chlorine in perchlorate exists in its most oxidized form, providing kinetic stability and resistance to further oxidation. As ClO_4_^–^ is a noncoordinating anion, it avoids forming coordination complexes with metal ions, which occur in the presence of Cl^–^, thereby negating the corrosion issue associated with Cl^–^ use. Perchlorate has a significantly higher concentration than phosphate in PBP, serving as a supporting electrolyte that nearly eliminates migration contributions to the mass transfer of electroactive species, enhances conductivity, and reduces *iR* drop. Two catalysts, IrO_*x*_ (a benchmarking catalyst)^46,47^ and Co(OH)_2_ (a widely studied nonprecious metal catalyst),^6,8^ along with a gold substrate, were studied in PBS and PBP electrolyte to elucidate the impact and assess the OER efficiency.

To assess chloride-containing PBS’s impact on OER performance measurement in electrocatalysts, we investigated three key aspects. First, we observed an excess in anodic current in PBS compared to PBP in which Cl^–^ is substituted with ClO_4_^–^. Second, we established that this excess current was unrelated to OER activity. Lastly, we proposed two potential mechanisms for this excess current: (electro)chemical oxidation of the catalyst, forming a soluble metal chloride salt, and chloride oxidation, resulting in hypochlorite formation. We show that both mechanisms occurred.

IrO_*x*_ of two different representative loadings, 18 and 100 μg cm^–2^, was first investigated using cyclic voltammetry (CV) on an RDE (Figure S1). The experiments were repeated three times, and average current densities at different potentials are shown (Figure 1a, 1b). IrO_*x*_ generally exhibit higher anodic currents in PBS compared to PBP (Figure 1a, 1b). At onset potential, 1.6 V vs RHE, current densities in PBS are 29% and 27% higher than in PBP for 18 and 100 μg cm^–2^ loadings, respectively (Figure 1a). At a larger overpotential, corresponding to 1.7 V vs RHE, current densities in PBS are 17% and 20% higher than in PBP for 18 and 100 μg cm^–2^ loadings, respectively (Figure 1b). Error bars represent the standard deviation of three replicates. The currents in the presence of Cl^–^ (PBS) are generally larger than in ClO_4_^–^ (PBP), and the extra charge consumed is shown below to be due to HCFR and corrosion. Our observations align with data from researchers aiming to enhance OER while suppressing HCFR in the presence of Cl^–^.^48^ The anodic curves of the same electrode (100 μg_IrOx_ cm^–2^) in PBS and PBP are shown (Figure 1c). At a slow scan rate of 5 mV s^–1^, the excess in anodic current in PBS compared to PBP is clearly observable and repeatable. The difference in current in the presence and absence of Cl^–^ is more pronounced with higher scan rate (Figure S3a, S3b). Nevertheless, a lower scan rate better represents the performance of the catalyst under steady-state conditions.

**Figure 1:**
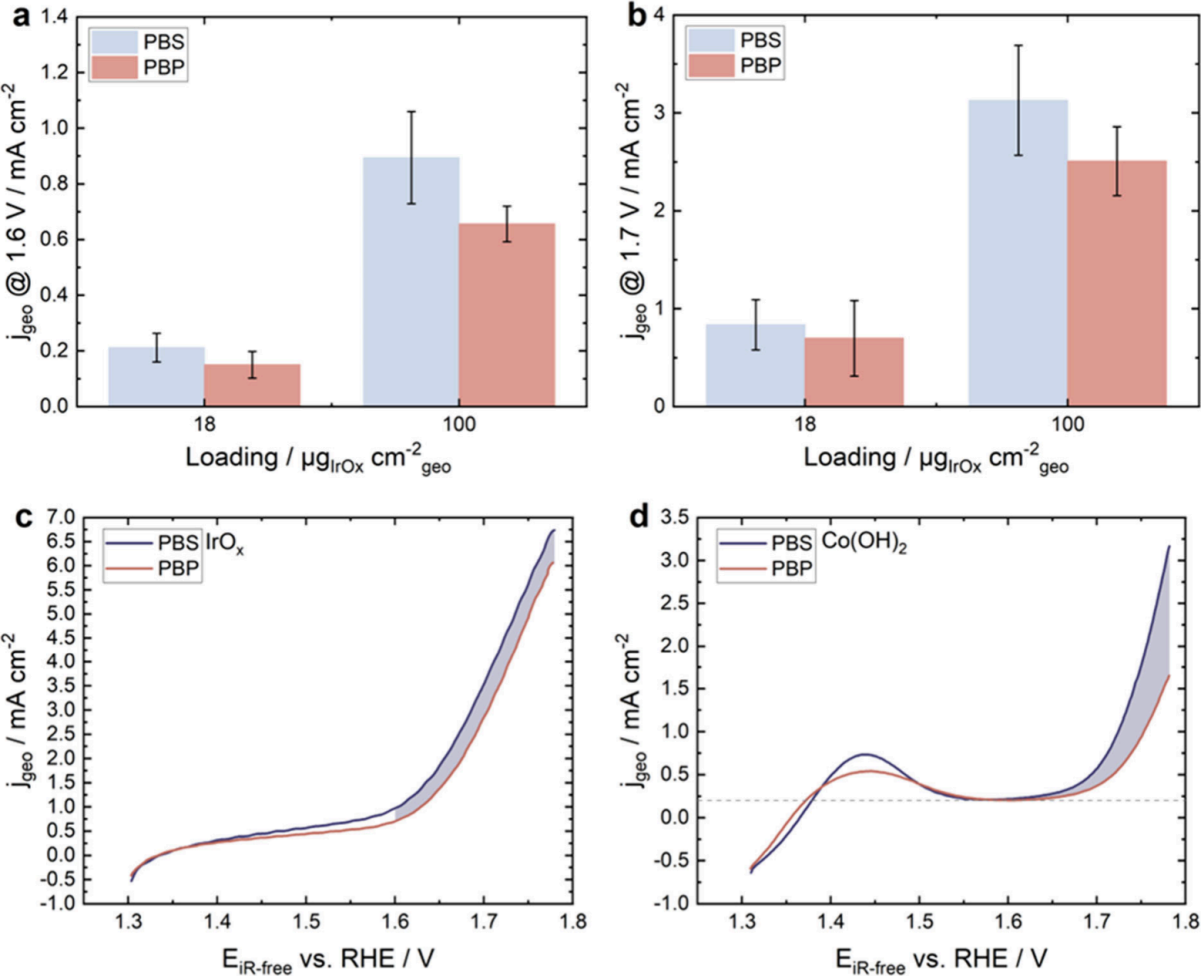
**Anodic polarization of IrO**_*x*_**and Co(OH)**_**2**_**in PBS and PBP.** Average current densities of IrO_*x*_ (3-runs) with different loadings at (a) 1.6 V forward vs RHE and (b) 1.7 V forward vs RHE in 1× PBS and 1× PBP. Anodic CV curves of (c) IrO_*x*_ with 100 μg cm^–2^ and (d) of Co(OH)_2_ in 1× PBS and 1× PBP. Potentials were corrected for solution resistance. CV curves were recorded at 5 mV s^–1^ in an Ar-saturated environment at 23 °C and pressure at 1600 rpm. The dotted lines represent baseline, and areas under anodic current after 1.6 V vs RHE are shaded. To avoid differences between electrode batches, anodic CV curves from the same electrode are shown. The applied potential and electrochemical current density were corrected for solution resistance and background current of the glassy carbon electrode (<10 μA cm^–2^) under Ar.

IrO_*x*_, a well-established corrosion-resistant OER catalyst,^49,50^ exhibits increased current indicative of chloride oxidation rather than complete OER in PBS. We further explored PBS’s impact on another catalyst, cobalt hydroxide (Co(OH)_2_), selected due to the prevalent focus on nonprecious metals as OER catalysts under neutral conditions.^6^ Co-based catalysts have garnered significant attention due to their supposed stability and high catalytic performance, and many recent highly cited publications in this domain have employed PBS as the electrolyte.^6,7,11^

A Co(OH)_2_ thin film was electrodeposited on a glassy carbon electrode, following literature protocols, detailed in the Supporting Information.^38,51^ Anodic CV curves of Co(OH)_2_ in PBS and PBP reveal a redox couple corresponding to Co^2+^/Co^3+^ oxidation, showcasing a quasi-reversible couple consistent with literature findings (Figure 1d).^8,52^ Comparing anodic curves of Co(OH)_2_ in PBS and PBP, the current density in PBS is significantly higher than that in PBP (Figures 1d, S3c, and S3b). This could be attributed to HCFR and oxidative corrosion of cobalt catalyst. Anodic curves of IrO_*x*_ and Co(OH)_2_ in PBS and PBP are shown for comparison as well (Figure 1c, 1d). The extra current densities in PBS (shaded in blue) for Co(OH)_2_ are higher than those of IrO_*x*_.

Rotating ring-disk electrode (RRDE) experiments were conducted to assess the anodic current contribution from OER in Ar-saturated electrolyte (Figure 2). The ring was held at a potential (0.3 V vs RHE) to measure the oxygen evolved at the disk via its reduction. To confirm limiting ORR behavior at 0.3 V vs RHE, a CV using the ring electrode in oxygen-saturated electrolyte was recorded (Figure S4). As discussed earlier, anodic currents in PBS exceed those in PBP for both IrO_*x*_ and Co(OH)_2_. For IrO_*x*_, the higher anodic current observed in PBS *does not* correlate with increased ring current, suggesting that excess disk current in PBS is not due to increased OER (Figure 2a). Similarly for Co(OH)_2_, the anodic current on the disk in PBS is significantly higher, but the ring current and consequently the OER activity of the central disk electrode are slightly lower in PBS (Figure 2b). Calculated OER Faradaic efficiency, with literature reference (details in the Supporting Information), indicates lower efficiency in PBS than in PBP. Specifically, for IrO_*x*_, the efficiencies are 57% in PBS and 72% in PBP. For Co(OH)_2_, the efficiencies are 36% in PBS and 80% in PBP. A Faradaic efficiency of 100% was not measured on the ring because of formation of gas bubbles which transport a fraction of the oxygen away from the ring.^53^ We assume this is unaffected by buffer choice. It is evident that the observed additional current in PBS is not attributed to OER but likely to HCFR. The differences in OER efficiencies in PBS and PBP are higher in Co(OH)_2_ than IrO_*x*_, indicating greater anodic current contribution from HCFR and potential catalyst corrosion on this catalyst.

**Figure 2:**
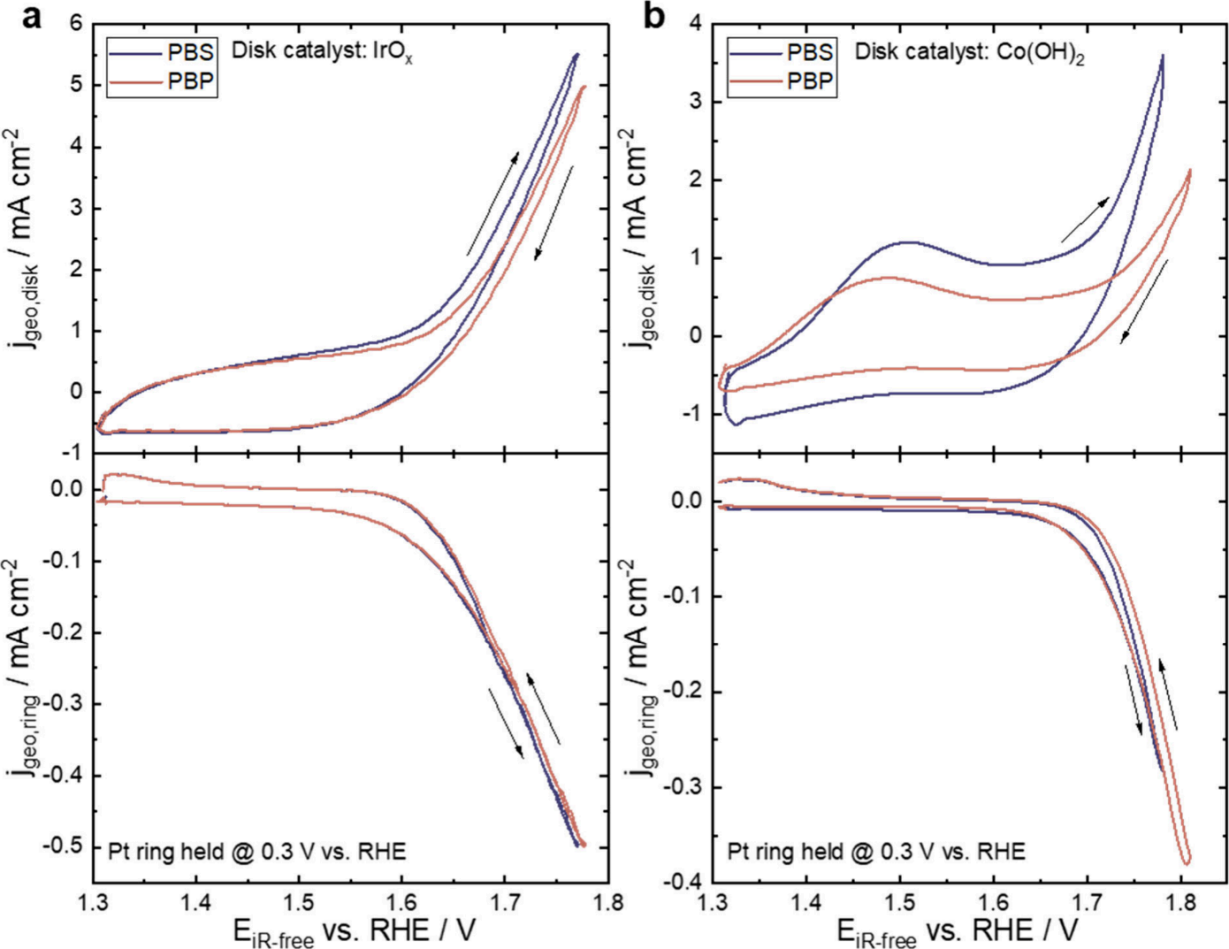
**RRDE experiments of IrO**_*x*_**and Co(OH)**_**2**_**in PBS and PBP.** RRDE measurements of (a) 100 μg cm^–2^ of IrO_*x*_ and (b) electrodeposited Co(OH)_2_ thin film on a glassy carbon substrate in 1× PBS and 1× PBP at 20 mV s^–1^ and 1600 rpm. Pt ring was held at 0.3 V vs RHE for oxygen reduction. Solution resistance and background were corrected.

We attempted to measure hypochlorite in the RRDE environment by holding the ring potential at 1.15 V vs RHE (a potential at which ORR would not occur but hypochlorite reduction is thermodynamically possible). However, this resulted in insufficient overpotential being applied to reduce hypochlorite at the ring due to its sluggish kinetics,^54^ rendering it unfeasible to assess hypochlorite production using RRDE (Figure S5).

Using eqs 1 and 2 and the known chloride concentration of the PBS buffer, we can estimate the *reversible* potential of the hypochlorite formation reactions. The reversible potential only aligns with the equilibrium potentials in eqs 1 and 2 when the hypochlorite concentration matches the chloride concentration. At the beginning of the reaction, the absence of hypochlorite causes a significant cathodic shift (>200 mV) in the reversible potential, as the denominators in eqs 1 and 2 are effectively zero. Consequently, hypochlorite quickly forms at low potentials to stabilize its concentration at the electrode surface. Deviations in the voltammetry are observed (Figures 1 and 2) at approximately 1.35 V, corresponding to an equilibrium hypochlorite concentration in the catalyst layer on the order of nM (Figure S11). On the RDE surface, formation of hypochlorite will be balanced by fast advection of the produced hypochlorite into the bulk solution. As the potential increases, the rate of hypochlorite production also rises, leading to higher steady-state concentrations of hypochlorite at the electrode surface.

As researchers report new materials using PBS as electrolyte, the exact nature of their interaction with chloride ion remains uncertain. An issue associated with chloride-containing electrolytes is the enhanced dissolution of materials under oxidizing conditions. For instance, platinum group metals (including iridium) show enhanced dissolution in sodium hypochlorite at room temperature with dissolution rates of 1–10 mg cm^–2^ h^–1^.^55^ Gold serves as both an (electrode) substrate and as catalyst in its own right.^56−58^ Hence, we highlight the performance of gold in PBS as an illustrative example of a material that is seemingly stable but susceptible to significant corrosion in the presence of chloride ions, even in mild pH conditions. Figure 3a depicts the voltammetry of gold as the central disk catalyst and a Pt ring held at 1.15 V vs RHE. Substantial anodic disk and cathodic ring currents are observed in PBS, while minimal currents are observed in both the disk and the ring in PBP. During anodic polarization of the central disk electrode, gold dissolution and subsequent deposition of gold in the ring occur *only in the presence of Cl*^**–**^ in PBS, but not in PBP containing ClO_4_^–^ (Figure 3b, 3c). This suggests selective gold dissolution in the presence of Cl^–^ when a potential is applied,^59^ attributing almost all observed anodic current in the central disk electrode in PBS to gold dissolution. Therefore, using gold as an electrode substrate or electrocatalyst is constrained by its dissolution in chloride-containing solutions. Moreover, it is advisable to avoid using PBS to assess the performance and mechanism of OER electrocatalysts, as it is challenging to decipher if (electro)chemical oxidation of the catalyst contributes to the observed anodic current.

**Figure 3:**
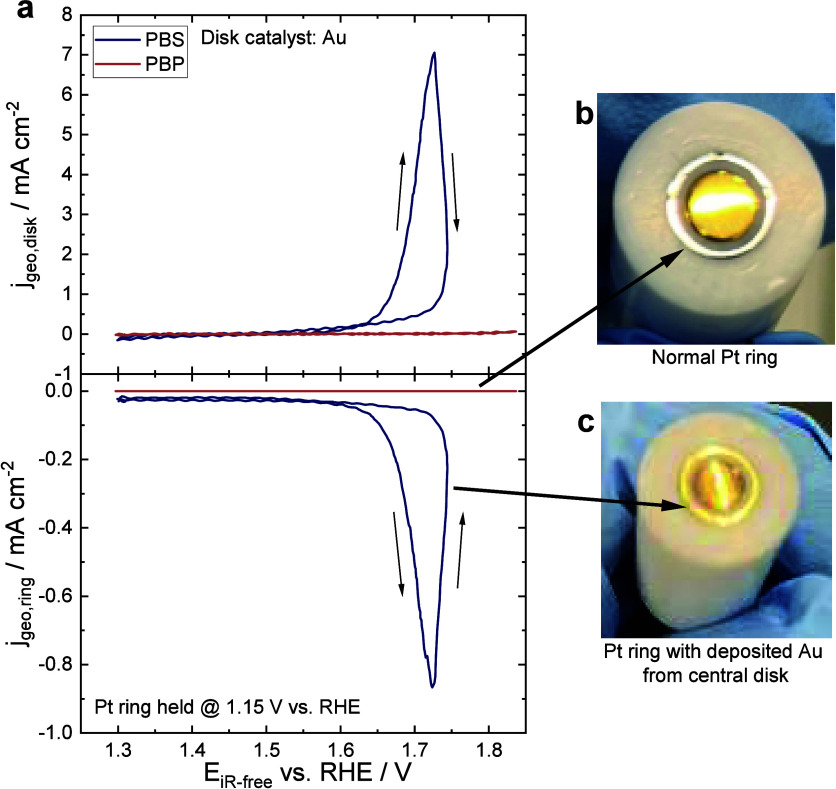
**RRDE experiments of Au in PBS and PBP.** (a) RRDE measurements of gold disk in 1× PBS and 1× PBP at 20 mV s^–1^ and 1600 rpm, with Pt ring held at 1.15 V vs RHE. Images of RRDE tip after OER in (b) 1× PBP and (c) 1× PBS. The greyish-white Pt ring was deposited with gold dissolved from the gold disk during anodic polarization in 1× PBS. Measurements were performed in an Ar-saturated environment at room temperature. Solution resistance and background were corrected.

From prior CV data, we deduce that a significant portion of the current possibly originated from chloride oxidation in PBS. To confirm hypochlorite production and quantify its amount, we conducted bulk electrolysis and measured hypochlorite levels using 3,3′,5,5′-tetramethyl benzidine (TMB). TMB, selected for its ability to detect low hypochlorite concentrations with excellent selectivity, undergoes rapid oxidation by hypochlorite or chlorite ions—products of chloride oxidation in PBS—to a blue charge transfer complex when TMB is in excess at pH 4 (Figure S6).^60,61^ Perchlorate ions in PBP, kinetically stable under ambient conditions, do not oxidize TMB. A UV/vis calibration curve at 650 nm was generated by reacting diluted NaClO standards with excess TMB (Figures S7, S8). The Faradaic efficiency for HCFR, *ε*_*HCFR*_, is calculated using eq 5.^38,41^
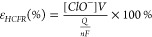
5where [ClO^–^] is the concentration of hypochlorite (mol dm^–3^) in the electrolyte determined from calibration, *V* is the total electrolyte volume (dm^3^), *Q* is the total charge delivered (C), *n* is the number of electron transfers (2 for HCFR), and *F* is Faraday’s constant (96485 C mol^–1^).

Chronopotentiometric curves for IrO_*x*_ and Co(OH)_2_ at 2 mA cm^–2^ (40 C cm^–2^ total charge) in PBS and PBP are presented (Figure 4a, 4b), with corresponding UV/vis spectra of the electrolytes containing excess TMB (Figure 4c). The observed potential increase over time is attributed to microscopic oxygen bubble shielding.^53^ A slightly larger potential is required in PBS compared to PBP for IrO_*x*_ (Figure 4a). However, a significant potential increase of about 117 mV is observed with Co(OH)_2_ in PBS after 2400 s, and the potential required in PBS is 110 mV higher than that required in PBP. The larger potential required in PBS for Co(OH)_2_ is attributed to HCFR and cobalt corrosion in the presence of Cl^–^, supported by the UV/vis spectra shown in Figure 4c. After IrO_*x*_ bulk electrolysis with excess TMB, the PBS electrolyte exhibits a typical spectrum of oxidized TMB due to the presence of ClO^–^ (peak at 650 nm). Similarly, after Co(OH)_2_ bulk electrolysis, a strong peak close to 650 nm is seen, although in this case, the main peak widens, and the peak absorbance shifts from 650 to 640 nm.

**Figure 4:**
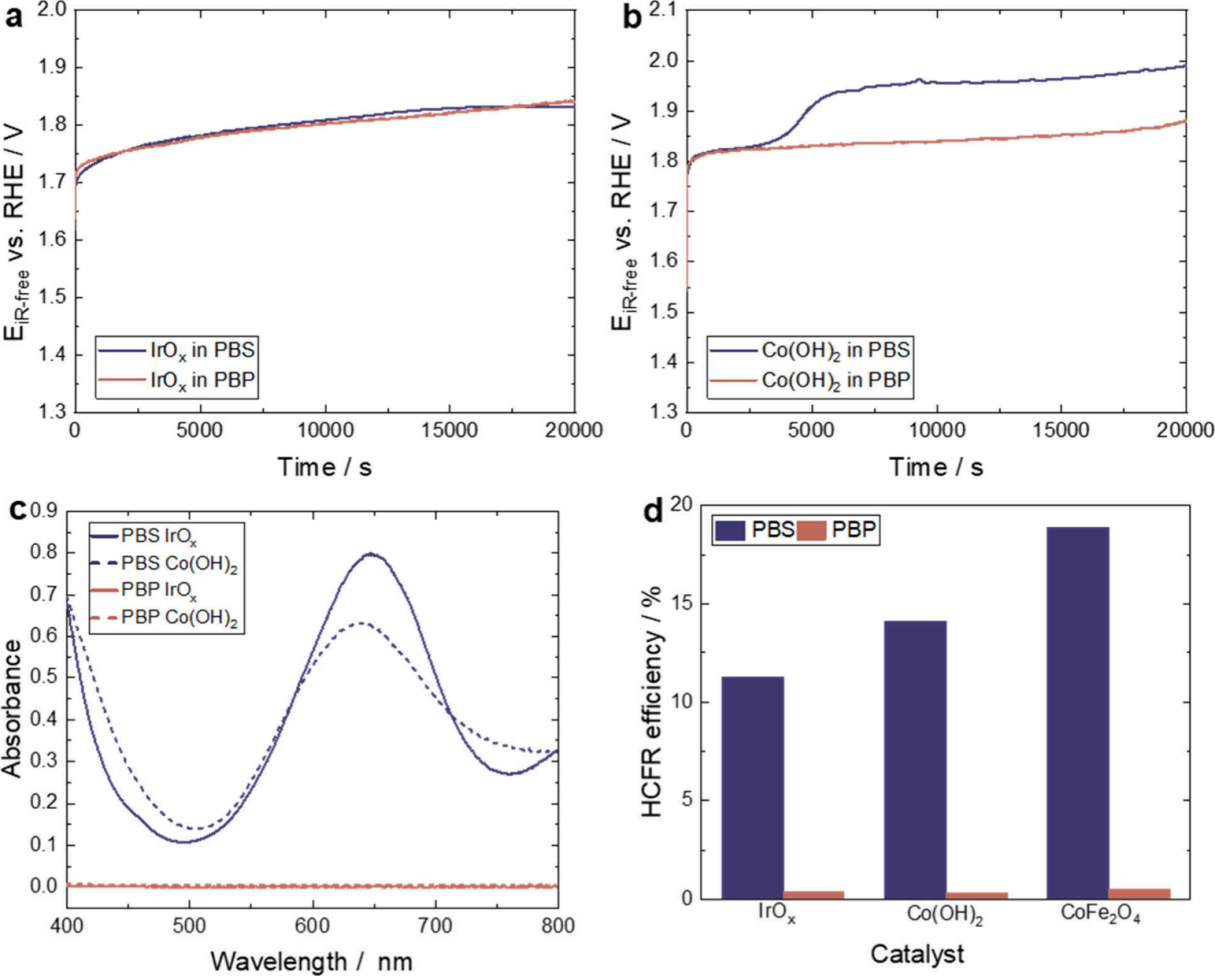
**Bulk electrolysis test and OER efficiency calculation.** Chronopotentiometric curves of (a) 100 μg cm^–2^ IrO_*x*_ and (b) Co(OH)_2_ at 2 mA cm^–2^ in 1× PBS and 1× PBP. Curves were recorded in an Ar-saturated environment at room temperature and pressure at 1600 rpm. (c) UV/vis absorption spectra of electrolytes after electrolysis with excess TMB in acetic buffer (pH 4.8). (d) Calculated HCFR efficiencies of different catalysts in PBS (blue) and PBP (red).

HCFR efficiencies, calculated from the total charge and amount of oxidized chlorine in the solution, are depicted in Figure 4d. In PBP, the non-HCFR efficiencies for all tested catalysts are inconsequential. The colorimetric method reveals HCFR efficiencies of 14% with IrO_*x*_ and 12% with Co(OH)_2_ in PBS, lower than RRDE measurements, which reveals non-OER Faradaic efficiencies of 15% with IrO_*x*_ and 44% with Co(OH)_2_. This is expected, as the colorimetric method does not consider current contribution from catalyst corrosion. Furthermore, some oxidized Cl^–^ could be lost as Cl_2_ into the gas phase during the experiment. The performance of cobalt iron oxide (CoFe_2_O_4_) is also examined, revealing a long-term HCFR efficiency of 19% in 1× PBS.

In conclusion, our study highlighted the overlooked impact of chloride oxidation in PBS on OER electrocatalyst measurements. We proposed PBP as a more suitable alternative, containing perchlorate ions to minimize absorptivity and ensure kinetic stability. We demonstrated excess anodic currents with IrO_*x*_ and Co(OH)_2_ catalysts in PBS buffer in contrast to PBP. RRDE experiments with both catalysts indicated that the excess currents in PBS were not OER-induced, revealing non-OER Faradaic efficiencies of 15% and 44% for IrO_*x*_ and Co(OH)_2_ respectively. We proposed two potential mechanisms for excess current in PBS: electrochemical oxidation of catalyst/substrate and oxidation of Cl^–^ forming ClO^–^. The former was demonstrated by gold substrate dissolution and subsequent deposition in the ring. Long-term bulk electrolysis with TMB indicated the latter, attributing 12–19% of anodic currents to HCFR in PBS depending on the catalysts.

This investigation sheds light on the potential misinterpretation of OER electrocatalyst performance in PBS, advocating against its use for assessing OER electrocatalysts’ performances and mechanisms. The presence of chloride ions complicates the assessment, hindering accurate understanding of intrinsic catalytic properties. Instead we suggest that a phosphate buffer composed of 0.1 M of each of Na_2_HPO_4_, NaH_2_PO_4_, and NaClO_4_, which we label as “(0.1 M)^3^ PBP, is a suitable alternative system. This system has a buffer pH of 7.2 and suitable buffer capacity to allow measurement at up to 10 mA cm^–2.^^18^ By including extra perchlorate salt, we increase the conductivity and decrease the issue of migration effects of the buffer molecules. If PBS is preferred over PBP, a meticulous investigation with proper attribution of anodic currents to HCFR via ClO^–^ quantification and catalyst corrosion via RRDE assessment is essential for accurate quantification.

## Supporting Information Available

Experimental methods; compositions of buffer choices; CV curves of IrO_*x*_ and Co(OH)_2_ catalysts at different scan rates; electrodeposition of Co(OH)_2_; limiting ORR behavior of Pt ring; RRDE experiments of IrO_*x*_ in PBS and PBP; schematic illustration of detecting hypochlorite ions through oxidation of TMB; UV/vis calibration of TMB and different concentrations of hypochlorite ions; UV/vis spectra of TMB in pure salt solutions (PDF)

## References

[ref1] Carmo M.; Fritz D. L.; Mergel J.; Stolten D. A Comprehensive Review on PEM Water Electrolysis. Int. J. Hydrog. Energy 2013, 38 (12), 4901–4934. 10.1016/j.ijhydene.2013.01.151.

[ref2] Ayers K. E.; Anderson E. B.; Capuano C.; Carter B.; Dalton L.; Hanlon G.; Manco J.; Niedzwiecki M. Research Advances towards Low Cost, High Efficiency PEM Electrolysis. ECS Trans. 2010, 33 (1), 3 10.1149/1.3484496.

[ref3] Tahir M.; Pan L.; Idrees F.; Zhang X.; Wang L.; Zou J.-J.; Wang Z. L. Electrocatalytic Oxygen Evolution Reaction for Energy Conversion and Storage: A Comprehensive Review. Nano Energy 2017, 37, 136–157. 10.1016/j.nanoen.2017.05.022.

[ref4] Zhang K.; Zou R. Advanced Transition Metal-Based OER Electrocatalysts: Current Status, Opportunities, and Challenges. Small 2021, 17 (37), 2100129 10.1002/smll.202100129. 34114334

[ref5] Cherevko S.; Geiger S.; Kasian O.; Kulyk N.; Grote J.-P.; Savan A.; Shrestha B. R.; Merzlikin S.; Breitbach B.; Ludwig A.; Mayrhofer K. J. J. Oxygen and Hydrogen Evolution Reactions on Ru, RuO2, Ir, and IrO2 Thin Film Electrodes in Acidic and Alkaline Electrolytes: A Comparative Study on Activity and Stability. Catal. Today 2016, 262, 170–180. 10.1016/j.cattod.2015.08.014.

[ref6] Li P.; Zhao R.; Chen H.; Wang H.; Wei P.; Huang H.; Liu Q.; Li T.; Shi X.; Zhang Y.; Liu M.; Sun X. Recent Advances in the Development of Water Oxidation Electrocatalysts at Mild pH. Small 2019, 15 (13), 1805103 10.1002/smll.201805103. 30773809

[ref7] Wang J.; Cui W.; Liu Q.; Xing Z.; Asiri A. M.; Sun X. Recent Progress in Cobalt-Based Heterogeneous Catalysts for Electrochemical Water Splitting. Adv. Mater. 2016, 28 (2), 215–230. 10.1002/adma.201502696. 26551487

[ref8] Zhang Y.; Wu C.; Jiang H.; Lin Y.; Liu H.; He Q.; Chen S.; Duan T.; Song L. Atomic Iridium Incorporated in Cobalt Hydroxide for Efficient Oxygen Evolution Catalysis in Neutral Electrolyte. Adv. Mater. 2018, 30 (18), 1707522 10.1002/adma.201707522. 29575370

[ref9] Yu J. M.; Song J.; Kim Y. K.; Oh J.; Kim K. Y.; Noh W. Y.; Byun W. J.; Lee J. U.; Yang C.; Jang J.-W.; Lee J. S.; Cho S. High-Performance Electrochemical and Photoelectrochemical Water Splitting at Neutral pH by Ir Nanocluster-Anchored CoFe-Layered Double Hydroxide Nanosheets. Nano Lett. 2023, 23 (11), 5092–5100. 10.1021/acs.nanolett.3c01024. 37212638

[ref10] Merrill M. D.; Dougherty R. C. Metal Oxide Catalysts for the Evolution of O2 from H2O. J. Phys. Chem. C 2008, 112 (10), 3655–3666. 10.1021/jp710675m.

[ref11] Dong Y.; Komarneni S. Strategies to Develop Earth-Abundant Heterogeneous Oxygen Evolution Reaction Catalysts for pH-Neutral or pH-Near-Neutral Electrolytes. Small Methods 2021, 5 (1), 2000719 10.1002/smtd.202000719. 34927809

[ref12] Surendranath Y.; Kanan M. W.; Nocera D. G. Mechanistic Studies of the Oxygen Evolution Reaction by a Cobalt-Phosphate Catalyst at Neutral pH. J. Am. Chem. Soc. 2010, 132 (46), 16501–16509. 10.1021/ja106102b. 20977209

[ref13] Nocera D. G. Personalized Energy: The Home as a Solar Power Station and Solar Gas Station. ChemSusChem 2009, 2 (5), 387–390. 10.1002/cssc.200900040. 19408259

[ref14] Han B.; Risch M.; Lee Y.-L.; Ling C.; Jia H.; Shao-Horn Y. Activity and Stability Trends of Perovskite Oxides for Oxygen Evolution Catalysis at Neutral pH. Phys. Chem. Chem. Phys. 2015, 17 (35), 22576–22580. 10.1039/C5CP04248H. 26271910

[ref15] Liang X.; Wang S.; Feng J.; Xu Z.; Guo Z.; Luo H.; Zhang F.; Wen C.; Feng L.; Wan C.; Titirici M.-M. Structural Transformation of Metal–Organic Frameworks and Identification of Electrocatalytically Active Species during the Oxygen Evolution Reaction under Neutral Conditions. Inorg. Chem. Front. 2023, 10 (10), 2961–2977. 10.1039/D2QI02436E.

[ref16] Liu Y.; Li P.; Wang Z.; Gao L. Shape–Preserved CoFeNi–MOF/NF Exhibiting Superior Performance for Overall Water Splitting across Alkaline and Neutral Conditions. Materials 2024, 17 (10), 2195 10.3390/ma17102195. 38793262 PMC11123414

[ref17] Sadaqat M.; Manzoor S.; Aman S.; Nisar L.; Najam-Ul-Haq M.; Shah A.; Shawky A. M.; Elhosiny Ali H.; Ashiq M. N.; Taha T. A. Defective Nickel Zirconium Oxide Mesoporous Bifunctional Electrocatalyst for Oxygen Evolution Reaction and Overall Water Splitting. Fuel 2023, 333, 126538 10.1016/j.fuel.2022.126538.

[ref18] Tariq I.; Ali A.; Haider A.; Iqbal W.; Asghar M. A.; Badshah A.; Mansoor M. A.; Nisar T.; Wagner V.; Abbas S. M.; Talat R. Nickel-Foam- and Carbon-Nanotubes-Fiber-Supported Bismuth Oxide/Nickel Oxide Composite; Highly Active and Stable Bifunctional Electrocatalysts for Water Splitting in Neutral and Alkaline Media. Energy Technol. 2024, 12 (4), 2301504 10.1002/ente.202301504.

[ref19] Yang S.; Liu X.; Li S.; Yue K.; Fan Y.; Yan Y.; Zhang W. Effects from Surface Structures of Manganese Phosphate on Electrocatalytic Water Oxidation. J. Phys. Chem. C 2024, 128 (20), 8181–8187. 10.1021/acs.jpcc.4c01930.

[ref20] Zhang K.; Jiang P.; Gu Q.; Leng Y.; Zhang P.; Li Z.; Li Y. Design of Cobalt-Iron Complex Sulfides Grown on Nickel Foam Modified by Reduced Graphene Oxide as a Highly Effect Bifunctional Electrocatalyst for Overall Water Splitting. Int. J. Energy Res. 2022, 46 (6), 7320–7333. 10.1002/er.7639.

[ref21] Zhang M.-K.; Chen W.; Xu M.-L.; Wei Z.; Zhou D.; Cai J.; Chen Y.-X. How Buffers Resist Electrochemical Reaction-Induced pH Shift under a Rotating Disk Electrode Configuration. Anal. Chem. 2021, 93 (4), 1976–1983. 10.1021/acs.analchem.0c03033. 33395265

[ref22] Kumeda T.; Sakaushi K. Joint Kinetic/In Situ Spectrometric Investigation of the Multielectron/Multiproton-Transfer-Based Adsorption Electrode Process of Phosphate Anions on the Ir(111) Surface across a Comprehensive pH Range. J. Phys. Chem. C 2023, 127 (21), 10341–10354. 10.1021/acs.jpcc.3c01287.

[ref23] Yu J.; Dai Y.; He Q.; Zhao D.; Shao Z.; Ni M. A Mini-Review of Noble-Metal-Free Electrocatalysts for Overall Water Splitting in Non-Alkaline Electrolytes. Mater. Rep. Energy 2021, 1 (2), 100024 10.1016/j.matre.2021.100024.

[ref24] Li P.; Zhao R.; Chen H.; Wang H.; Wei P.; Huang H.; Liu Q.; Li T.; Shi X.; Zhang Y.; Liu M.; Sun X. Recent Advances in the Development of Water Oxidation Electrocatalysts at Mild pH. Small 2019, 15 (13), 1805103 10.1002/smll.201805103. 30773809

[ref25] Dong Y.; Komarneni S. Strategies to Develop Earth-Abundant Heterogeneous Oxygen Evolution Reaction Catalysts for pH-Neutral or pH-Near-Neutral Electrolytes. Small Methods 2021, 5 (1), 2000719 10.1002/smtd.202000719. 34927809

[ref26] Wang J.; Cui W.; Liu Q.; Xing Z.; Asiri A. M.; Sun X. Recent Progress in Cobalt-Based Heterogeneous Catalysts for Electrochemical Water Splitting. Adv. Mater. 2016, 28 (2), 215–230. 10.1002/adma.201502696. 26551487

[ref27] Li G.; Yang F.; Che S.; Liu H.; Chen N.; Qian J.; Yu C.; Jiang B.; Liu M.; Li Y. Mo-Doped CoP Nanoparticles Anchored on Porous Co-N-C Framework as an Efficient Bifunctional Electrocatalyst for pH-Universal Water Splitting. J. Mater. Sci. Technol. 2023, 166, 58–66. 10.1016/j.jmst.2023.05.022.

[ref28] Pu Y.; Wang L.; Jia L.; Li X.; Lu W.; Huang L. Identifying the *in Situ* Protection Role of MnO2 Nanosheets on Co Oxide for Superior Water Oxidation. Appl. Surf. Sci. 2023, 636, 157647 10.1016/j.apsusc.2023.157647.

[ref29] Feng T.; Yu J.; Yue D.; Song H.; Tao S.; Waterhouse G. I. N.; Lu S.; Yang B. Defect-Rich Ruthenium Dioxide Electrocatalyst Enabled by Electronic Reservoir Effect of Carbonized Polymer Dot for Remarkable pH-Universal Oxygen Evolution. Appl. Catal. B Environ. 2023, 328, 122546 10.1016/j.apcatb.2023.122546.

[ref30] Balqis F.; Irmawati Y.; Geng D.; Nugroho F. A. A.; Sumboja A. Nanostructured Ball-Milled Ni–Co–Mn Oxides from Spent Li-Ion Batteries as Electrocatalysts for Oxygen Evolution Reaction. ACS Appl. Nano Mater. 2023, 10.1021/acsanm.3c02092.

[ref31] Wang X.; Wang Z.; Cao Y.; Liu X.; Zhou L.; Shi J.; Guo B.; Li D.; Ye R.; Zhao Z. A Facile Synthesis of Hierarchical CoFe2O4 Nanosheets for Efficient Oxygen Evolution in Neutral Medium. J. Solid State Chem. 2024, 331, 124553 10.1016/j.jssc.2024.124553.

[ref32] Safdar M.; Iftikhar M.; Rashid S.; Awais M.; Iqbal A.; Bilal A.; Aslam S.; Mirza M. Synthesis and Investigation of Catalytic HER/OER Performances of Al2SSe in Alkaline/Acidic Media and Water Detoxification Behavior. Int. J. Hydrog. Energy 2024, 50, 107–117. 10.1016/j.ijhydene.2023.07.252.

[ref33] Tian L.; Zhong D.; Zhao T.; Liu Y.; Hao L.; Fang Q.; Lang X.; Zhao X.; Hao G.; Liu G.; Li J.; Zhao Q. Oxygen-Vacancy-Rich Co3O4@Fe-B-O Heterostructure for Efficient Oxygen Evolution Reaction in Alkaline and Neutral Media. J. Colloid Interface Sci. 2023, 646, 452–460. 10.1016/j.jcis.2023.05.042. 37207426

[ref34] Bard A. Standard Potentials in Aqueous Solution; Routledge, 2017.

[ref35] Karlsson R. K. B.; Cornell A. Selectivity between Oxygen and Chlorine Evolution in the Chlor-Alkali and Chlorate Processes. Chem. Rev. 2016, 116 (5), 2982–3028. 10.1021/acs.chemrev.5b00389. 26879761

[ref36] Choi S.; Choi W. I.; Lee J.-S.; Lee C. H.; Balamurugan M.; Schwarz A. D.; Choi Z. S.; Randriamahazaka H.; Nam K. T. A Reflection on Sustainable Anode Materials for Electrochemical Chloride Oxidation. Adv. Mater. 2023, 35 (43), 2300429 10.1002/adma.202300429. 36897816

[ref37] Choi S.; Choi W. I.; Lee J.-S.; Lee C. H.; Balamurugan M.; Schwarz A. D.; Choi Z. S.; Randriamahazaka H.; Nam K. T. A Reflection on Sustainable Anode Materials for Electrochemical Chloride Oxidation. Adv. Mater. 2023, 35 (43), 2300429 10.1002/adma.202300429. 36897816

[ref38] Okada T.; Abe H.; Murakami A.; Shimizu T.; Fujii K.; Wakabayashi T.; Nakayama M. A Bilayer Structure Composed of Mg|Co-MnO2 Deposited on a Co(OH)2 Film to Realize Selective Oxygen Evolution from Chloride-Containing Water. Langmuir 2020, 36 (19), 5227–5235. 10.1021/acs.langmuir.0c00547. 32347730

[ref39] Hayes M.; Kuhn A. T. The Preparation and Behaviour of Magnetite Anodes. J. Appl. Electrochem. 1978, 8 (4), 327–332. 10.1007/BF00612686.

[ref40] Littauer E. E.; Shreir L. L. Anodic Oxidation of Pb2+ → PbO2 in Chloride Solutions. Electrochim. Acta 1967, 12 (5), 465–474. 10.1016/0013-4686(67)80016-1.

[ref41] Abe H.; Murakami A.; Tsunekawa S.; Okada T.; Wakabayashi T.; Yoshida M.; Nakayama M. Selective Catalyst for Oxygen Evolution in Neutral Brine Electrolysis: An Oxygen-Deficient Manganese Oxide Film. ACS Catal. 2021, 11 (11), 6390–6397. 10.1021/acscatal.0c05496.

[ref42] McCafferty E. Introduction to Corrosion Science; Springer Science & Business Media, 2010.

[ref43] Komiya H.; Shinagawa T.; Takanabe K. Electrolyte Engineering for Oxygen Evolution Reaction Over Non-Noble Metal Electrodes Achieving High Current Density in the Presence of Chloride Ion. ChemSusChem 2022, 15 (19), e202201088 10.1002/cssc.202201088. 35921042 PMC9804667

[ref44] Haq T. ul; Arooj M.; Tahir A.; Haik Y. SOx Functionalized NiOOH Nanosheets Embedded in Ni(OH)2 Microarray for High-Efficiency Seawater Oxidation. Small 2024, 20 (18), 2305694 10.1002/smll.202305694. 38078786

[ref45] Dresp S.; Dionigi F.; Klingenhof M.; Strasser P. Direct Electrolytic Splitting of Seawater: Opportunities and Challenges. ACS Energy Lett. 2019, 4 (4), 933–942. 10.1021/acsenergylett.9b00220.

[ref46] McCrory C. C. L.; Jung S.; Peters J. C.; Jaramillo T. F. Benchmarking Heterogeneous Electrocatalysts for the Oxygen Evolution Reaction. J. Am. Chem. Soc. 2013, 135 (45), 16977–16987. 10.1021/ja407115p. 24171402

[ref47] McCrory C. C. L.; Jung S.; Ferrer I. M.; Chatman S. M.; Peters J. C.; Jaramillo T. F. Benchmarking Hydrogen Evolving Reaction and Oxygen Evolving Reaction Electrocatalysts for Solar Water Splitting Devices. J. Am. Chem. Soc. 2015, 137 (13), 4347–4357. 10.1021/ja510442p. 25668483

[ref48] Abe H.; Kobayakawa T.; Maruyama H.; Wakabayashi T.; Nakayama M. Thin Film Coating of Mg-Intercalated Layered MnO2 to Suppress Chlorine Evolution at an IrO2 Anode in Cathodic Protection. Electrocatalysis 2019, 10 (2), 195–202. 10.1007/s12678-019-0509-3.

[ref49] Quinson J. Iridium and IrOx Nanoparticles: An Overview and Review of Syntheses and Applications. Adv. Colloid Interface Sci. 2022, 303, 102643 10.1016/j.cis.2022.102643. 35334351

[ref50] Wu W.; Chen Z. Iridium Coating: Processes, Properties and Application. Part I: Processes for Protection in High-Temperature Environments against Oxidation and Corrosion. Johns. Matthey Technol. Rev. 2017, 61 (1), 16–28. 10.1595/205651317X693606.

[ref51] Kong L.-B.; Liu M.-C.; Lang J.-W.; Liu M.; Luo Y.-C.; Kang L. Porous Cobalt Hydroxide Film Electrodeposited on Nickel Foam with Excellent Electrochemical Capacitive Behavior. J. Solid State Electrochem. 2011, 15 (3), 571–577. 10.1007/s10008-010-1125-6.

[ref52] Lin X.; Li H.; Musharavati F.; Zalnezhad E.; Bae S.; Cho B.-Y.; Hui O. K. S. Synthesis and Characterization of Cobalt Hydroxide Carbonate Nanostructures. RSC Adv. 2017, 7 (74), 46925–46931. 10.1039/C7RA09050A.

[ref53] Hartig-Weiss A.; Tovini M. F.; Gasteiger H. A.; El-Sayed H. A. OER Catalyst Durability Tests Using the Rotating Disk Electrode Technique: The Reason Why This Leads to Erroneous Conclusions. ACS Appl. Energy Mater. 2020, 3 (11), 10323–10327. 10.1021/acsaem.0c01944.

[ref54] Vos J. G.; Koper M. T. M. Measurement of Competition between Oxygen Evolution and Chlorine Evolution Using Rotating Ring-Disk Electrode Voltammetry. J. Electroanal. Chem. 2018, 819, 260–268. 10.1016/j.jelechem.2017.10.058.

[ref55] Speight J. G. Ullmann’s Encyclopedia of Industrial Chemistry. Pet. Sci. Technol. 1999, 17 (3–4), 445 10.1080/10916469908949727.

[ref56] Mai H. D.; Le V. C. T.; Yoo H. Effective Fabrication and Electrochemical Oxygen Evolution Reaction Activity of Gold Multipod Nanoparticle Core–Cobalt Sulfide Shell Nanohybrids. ACS Appl. Nano Mater. 2019, 2 (2), 678–688. 10.1021/acsanm.8b01689.

[ref57] Kim S.; Cho M.; Lee Y. Iridium Oxide Dendrite as a Highly Efficient Dual Electro-Catalyst for Water Splitting and Sensing of H2O2. J. Electrochem. Soc. 2017, 164 (5), B3029 10.1149/2.0061705jes.

[ref58] Bhartiya P. K.; Srivastava M.; Mishra D. Chiral-Induced Enhanced Electrocatalytic Behaviour of Cysteine Coated Bifunctional Au–Ni Bilayer Thin Film Device for Water Splitting Application. Int. J. Hydrog. Energy 2022, 47 (100), 42160–42170. 10.1016/j.ijhydene.2021.08.219.

[ref59] Diaz M. A.; Kelsall G. H.; Welham N. J. Electrowinning Coupled to Gold Leaching by Electrogenerated Chlorine: I. Au(III) Au(I)/Au Kinetics in Aqueous Cl2/Cl– Electrolytes. J. Electroanal. Chem. 1993, 361 (1), 25–38. 10.1016/0022-0728(93)87035-T.

[ref60] Guo Y.; Ma Q.; Cao F.; Zhao Q.; Ji X. Colorimetric Detection of Hypochlorite in Tap Water Based on the Oxidation of 3,3′,5,5′-Tetramethyl Benzidine. Anal. Methods 2015, 7 (10), 4055–4058. 10.1039/C5AY00735F.

[ref61] Yamaoka H.; Nakayama-Imaohji H.; Horiuchi I.; Yamasaki H.; Nagao T.; Fujita Y.; Maeda H.; Goda H.; Kuwahara T. Tetramethylbenzidine Method for Monitoring the Free Available Chlorine and Microbicidal Activity of Chlorite-based Sanitizers under Organic-matter-rich Environments. Lett. Appl. Microbiol. 2016, 62 (1), 47–54. 10.1111/lam.12506. 26460606

