# Supplementary material for: Avoid Using Phosphate Buffered Saline (PBS) as an Electrolyte for Accurate OER Studies
Source: ACS Energy Lett. 2024 Jul 18;9(8):3939–46. doi: 10.1021/acsenergylett.4c01589 (PMC11320652; doi:10.1021/acsenergylett.4c01589)
Supplement: Supplementary file 1 — nz4c01589_si_001.pdf [file nz4c01589_si_001.pdf]

Supporting Information

Avoid using phosphate buffered saline (PBS) as an Electrolyte for Accurate OER Studies

*Haiyi Wang, Xiaoqian Lin, and Anthony R. Kucernak**

Department of Chemistry, Imperial College London, White City, London, UK, W12 0BZ

*Corresponding author. Email: anthony@imperial.ac.uk

Section 1: Experimental methods

Chemicals. An ALNOCHROMIX™ solution comprising ALNOCHROMIX™ crystals (Merck) in 95% concentrated sulfuric acid (AnalaR NORMAPUR, VWR Chemicals) was used for glassware cleaning. Ultrapure water (18.2 MΩ cm, Merck Millipore Milli-Q) with total organic carbon (TOC) < 10 ppb was used for washing and, catalyst ink and electrolyte preparation. PBS (Phosphate Buffered Saline, 10× Solution, Fisher BioReagents) was used as stock for 1× PBS electrolyte preparation. PBP (Phosphate buffered perchlorate) was prepared from sodium phosphate dibasic (ReagentPlus®, ≥99.0%, Sigma-Aldrich), potassium dihydrogen phosphate (ACS reagent, ≥99.0%, Sigma-Aldrich), and sodium perchlorate monohydrate (ACS reagent, Sigma-Aldrich). Iridium (IV) oxide (Premion™, Ir 84.5% min, Thermo Scientific Chemicals), Nafion™ perfluorinated resin solution (5 wt. %, Sigma-Aldrich), and 2-propanol (AnalaR NORMAPUR ACS, Reag. Ph. Eur. analytical reagent, VWR Chemicals) were used for catalyst ink preparation. Cobalt (II) nitrate hexahydrate (Sigma-Aldrich) was used for cobalt hydroxide electrodeposition. Cobalt iron oxide, $CoFe_2O_4$, was used for chronopotentiometric study (Sigma-Aldrich). Sodium hypochlorite solution (6-14% active chlorine EMPLURA®), 3,3′,5,5′-Tetramethylbenzidine (≥99%, TMB), acetic acid (puriss. p.a., ACS reagent, reag. ISO, reag. Ph. Eur., ≥99.8%), and sodium acetate trihydrate were purchased from Sigma-Aldrich for hypochlorite calibration and quantification. Oxygen UltraPure Plus (5.8N purity)) and argon BIP Plus (6.0 purity) were purchased from Air Products.

Catalyst Preparation. Iridium oxide catalyst ink solutions of different concentrations were prepared by adding IrO_x into solution of isopropanol and Milli-Q water (3:1 ratio), followed by

sonication for 30 minutes at 27 ˚C. Then, 5 wt. % Nafion was added and the ionomer to catalyst weight ratio was 0.2 (Table S1). The concentrated ink solutions were sonicated for 1 hour at 15 ˚C. Inks that were drop-casted onto the WE were dried at 200 rpm for 5 mins, followed by 500 rpm for 20 mins. $CoFe_2O_4$ catalyst ink solution was prepared using the same formulation as the IrO_x ink solution.

Final WE loading / µg cm^{-2}	IrO_x / mg	IPA+H_2O solution / mL	Nafion 5 wt. % / µL	Ink concentration / mg_{IrOx} cm^{-3}	Volume of ink drop-casted / µL
18	1.8	3	0.4	0.60	6
100	6	3	26.1	1.98	9.91

Table S1. Catalyst ink formulation.

A thin film $Co(OH)_2$ was prepared according to reference by applying a constant potential of -0.542 V against the reference electrode (MMS) in 0.1 M $Co(NO_3)_2$ solution at 1600 rpm.[1,2] The charge density delivered was 43 mC cm^{-2}. Assuming all charges delivered were used for electrodeposition, the WE was loaded with 122 µg cm^{-2} cobalt hydroxide. The WE was then rinsed thoroughly with Milli-Q water and left at ambient conditions to dry overnight.

Electrochemical Apparatus and Measurements. To eliminate any impurities, such as organic compounds, metals, and salts on the surface, all glassware that came into contact with the electrolyte was washed with Milli-Q water and then immersed in the Nochcromix bath overnight. Following washing with flowing Milli-Q water to get rid of any Nochromix residue, the glassware was boiled six times for one hour each time to get rid of any remaining acid on the surface. When not in use, the glassware was kept in Milli-Q water, and a monthly cleaning process was conducted.

Before each electrochemical testing, the cell was rinsed with Milli-Q water three times to remove any airborne pollutants.

A conventional three-electrode assembly which was connected to a potentiostat (Metrohm Autolab) was used. Saturated mercury-mercurous sulphate (MMS) was used as the reference electrode (RE) in the Luggin Capillary compartment, and a graphite rod was used as the counter electrode (CE) in another compartment separated by a frit. Gold and platinum were not used as CE to avoid potential dissolution into the solution which would interfere with the working electrode (WE). Potential was converted to RHE scale using equation S1.

$$\text{Equation S1} \quad E_{RHE} = E_{Hg/Hg_2SO_4} + 0.7 + 0.059 \times pH$$

For RDE experiments, glassy carbon RDE tip (5.0 mm disk OD, 12.0 mm OD PTFE shroud) from Pine Research Instrumentation was used as the WE. For RRDE experiments, platinum ring assembly (15.0 mm OD PTFE shroud, Ring OD = 7.50 mm, Ring ID = 6.50 mm, Disk OD = 5.0 mm) with glassy carbon insert was used as the WE. OER efficiency was calculated from the following equation:[3]

$$\varepsilon = \frac{2i_r}{i_d N}$$

where i_r is the measured ring current, i_d is the disk current, and N is the collection efficiency.

1× PBS electrolyte was prepared by diluting 10× PBS stock solution using Milli-Q water, and 1× PBP was prepared by dissolving slats in Milli-Q water (Table S2). pH values of the solutions

were measured using a pH meter (Thermo Scientific Orion ROSS Sure-Flow Combination pH probe, calibrated monthly).

Salts in PBS (1×)	Concentration / mmol dm^{-3}	Conductivity / mS cm^{-1}	Salts in PBP (1×)	Concentration / mmol dm^{-3}	Conductivity / mS cm^{-1}
NaCl	137	13.75	$NaClO_4$	139.7	12.55
KCl	2.7				
Na_2HPO_4	10		Na_2HPO_4	10	
KH_2PO_4	1.8		KH_2PO_4	1.8	

Table S2. Common compositions of PBS (1×) and PBP (1×).

Electrochemical characterization was performed in either PBS (1×) or PBP (1×) electrolytes which were degassed using argon for 30 mis at room temperature. Electrochemical conditioning was performed by cycling 3 times from 1.3-1.80 VS RHE at a scan rate of 100 mVs^{-1}. Cyclic voltammetry was performed at scan rates of 30, 20, 10, and 5 mVs^{-1}. To correct for uncompensated solution resistance, the electrochemical impedance spectrum were obtained by sweeping a 10 mV_{RMS} sinusoidal modulation from 10 kHz to 0.1 Hz with the potential held at 1.59 V vs. RHE. The solution background from the glassy carbon substrate in 1×PBS is negligible compared to the substrate loaded with the electrocatalyst (Fig. S10). Nonetheless, all recorded CVs have been corrected by subtracting the solution background due to the substrate.

Hypochlorite Detection. The faradaic efficiency of HCFR was determined by performing bulk electrolysis and quantifying the amount of hypochlorite formation. A 5 mM TMB stock solution was prepared from dissolving TMB in Milli-Q water and ethanol mixture (50 : 50 v/v).[4] UV-vis calibration (Thermo Scientific Evolution 220 UV-Visible Spectrophotometers) was done by

adding acetic buffer (375 μL, 0.4 M, pH 4.8) and excess TMB solutions into different concentrations of sodium hypochlorite solutions. Milli-Q water was added to bring the volume to 4 mL.[4]

Bulk electrolysis was performed at constant current density of 2 mA cm^{-2} in 1× PBS or 1× PBP solutions at 1600 rpm. A constant charge density of 40 C cm^{-2} was delivered. Acetic buffer (375 μL, 0.4 M, pH 4.8) and TMB (100 μL, 5 mM) were added to 3 mL of the electrolyte after electrolysis. The solutions were incubated for 3 minutes before obtaining UV/Vis absorption spectra. Hypochlorite concentrations were determined from the calibration curve.

Section 2: Supplementary data

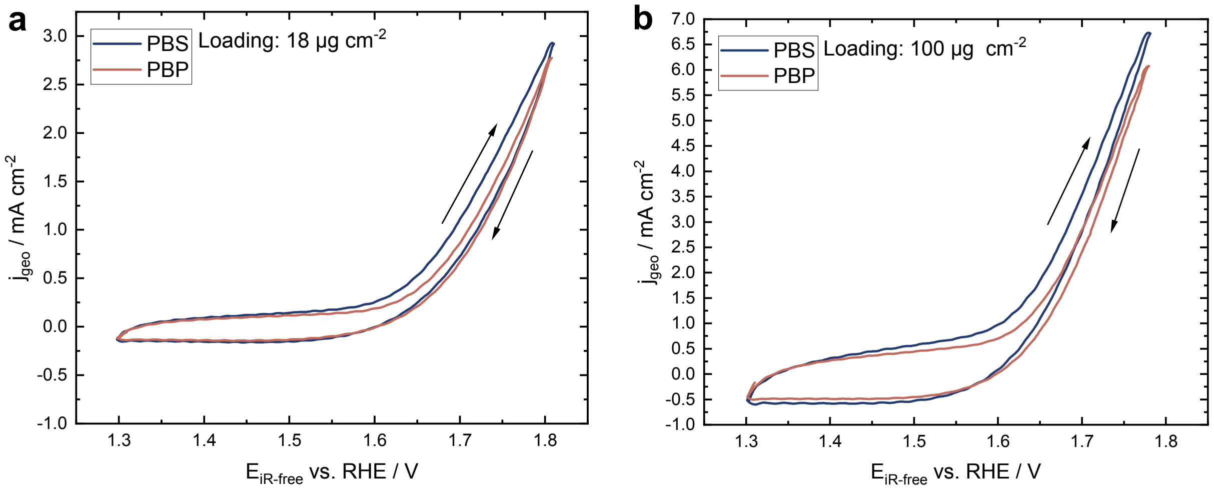

Figure S1. Illustrations of performance of IrO_x in PBS and PBP at different loadings. CVs of IrO_x were recorded at scan rate 20 mV s^{-1} in an Ar-saturated environment (2500 rpm) with loadings (a) 18 μg cm^{-2}, (b) 100 μg cm^{-2}. CVs of the same electrode done separately in PBP and PBS were shown. Solution resistance and background under Ar were used to correct the OER currents.

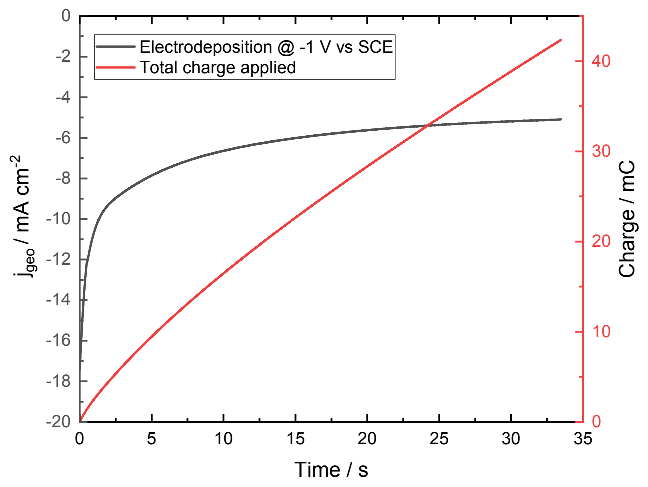

Figure S2. Chronoamperometric curve for electrodeposition of $Co(OH)_2$. The total charge density delivered was 43 mC. The deposition was carried out in 0.1 M $Co(NO_3)_2$ at 1600 rpm.

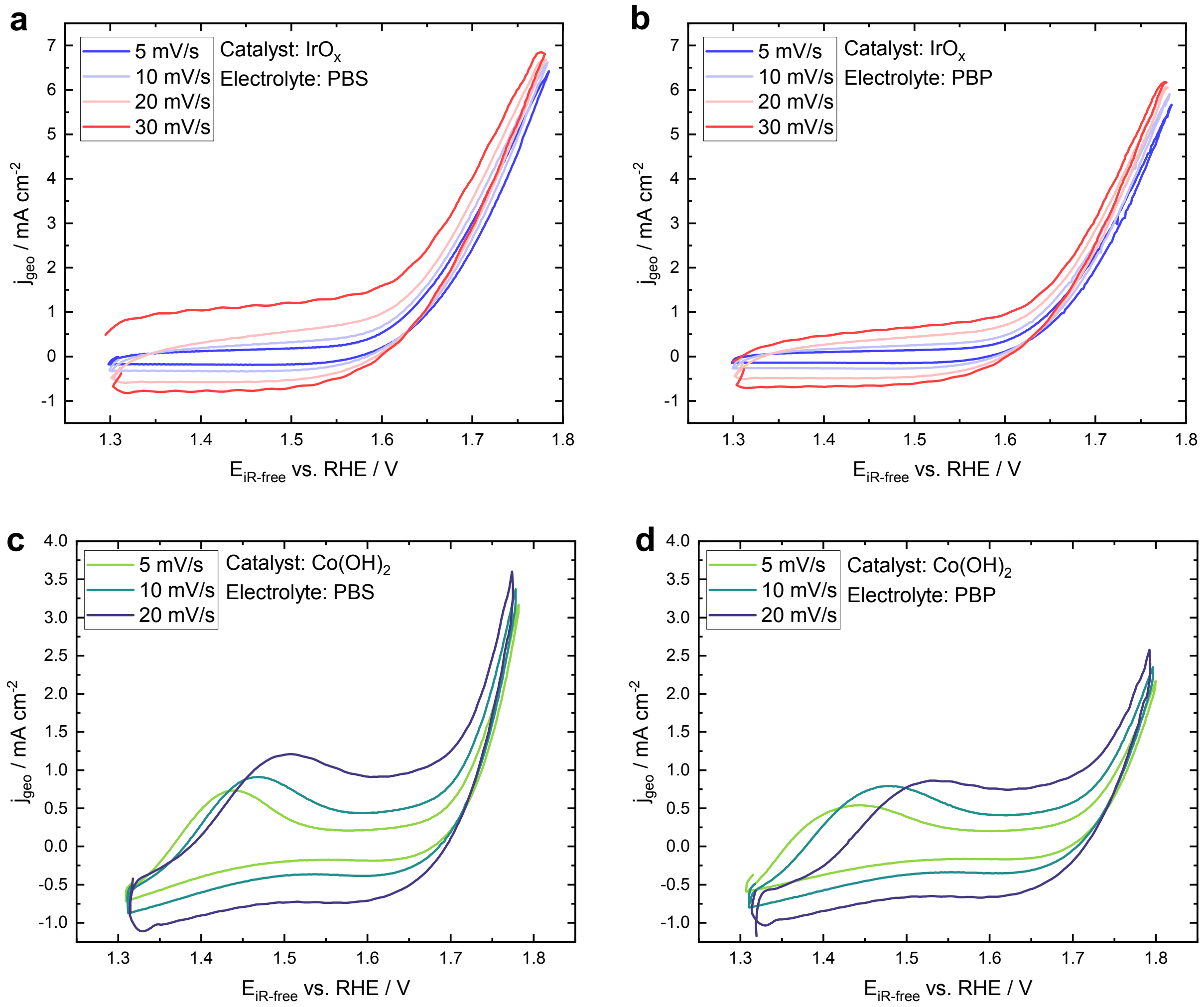

Figure S3. CV curves of IrO_x and $Co(OH)_2$ using different scan rates in PBS and PBP. CV curves of IrO_x with 100 μg cm^{-2} in (a) 1× PBS and (b) 1× PBP; CV curves of $Co(OH)_2$ in (a) 1× PBS and (b) 1× PBP. Data were recorded in an Ar-saturated environment at room temperature and pressure at 1600 rpm. To avoid differences between electrode batches, the same electrode was used. Solution resistance and background under Ar were corrected.

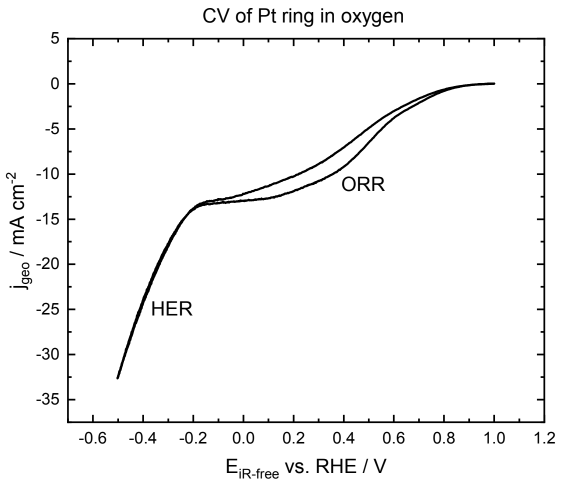

Figure S4. CV of platinum ring in an oxygen-saturated environment. The CV was recorded at 1600 rpm. 0.3 V vs. RHE was chosen to apply to the ring to perform reduction reactions for RRDE experiments. The oxygen reduction reaction (ORR) occurred within the potential range of -0.2 to 0 V vs. RHE.

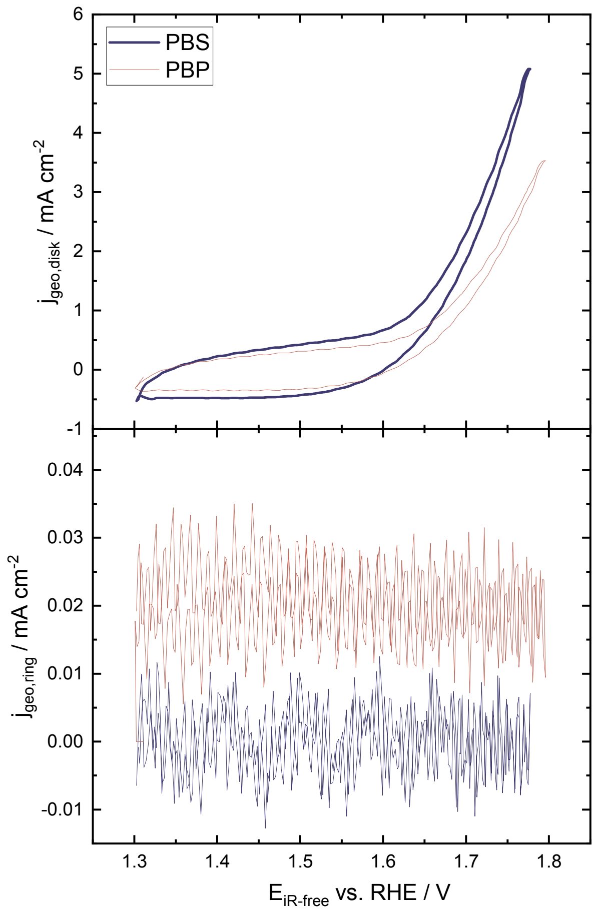

Figure S5. RRDE experiments of IrO_x in PBS and PBP. CVs were recorded with 100 µg cm^{-2} IrO_x drop-casted on glassy carbon disk and Pt ring was held at 1.15 V vs. RHE for hypochlorite oxidation in 1× PBS and 1× PBP respectively. CVs were recorded in an Ar-saturated environment at room temperature and pressure at 1600 rpm, and were background corrected. Solution resistance was also corrected.

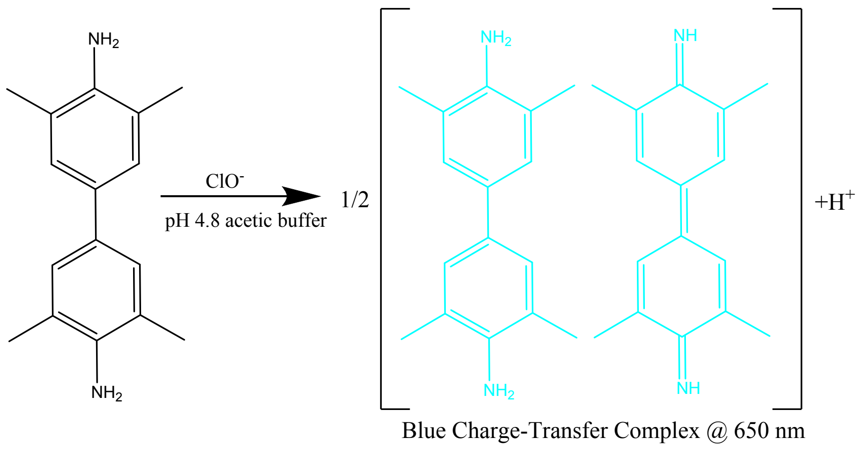

Figure S6. A schematic illustration depicts the colorimetric detection of hypochlorite ions through the oxidation of TMB at pH 4.8.

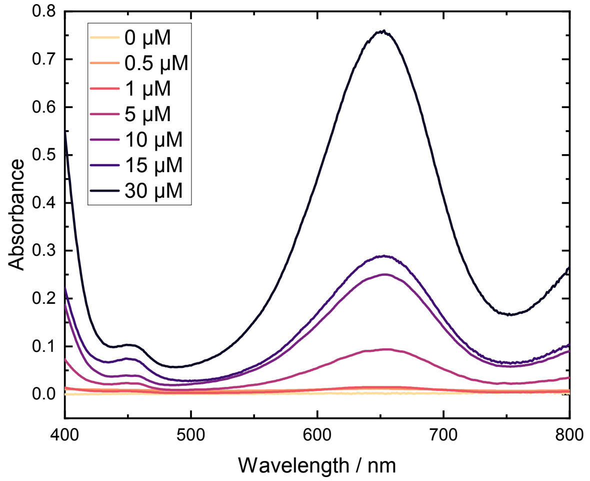

Figure S7. UV/Vis absorption spectra of TMB in pH 4.8 (acetic buffer) with different concentrations of ClO^-.

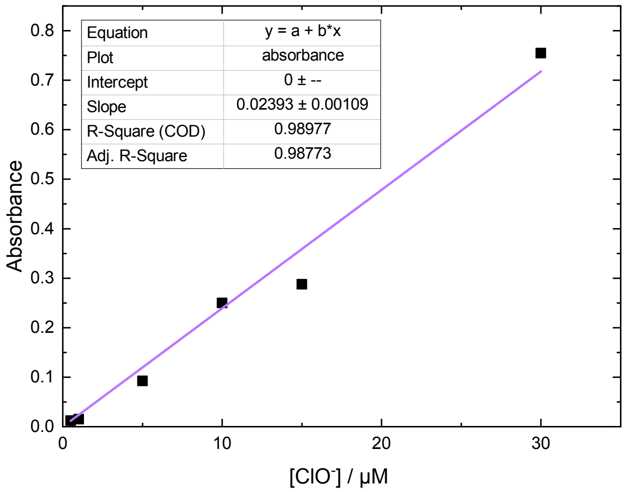

Figure S8. Linear relationship between peak absorbance at 650 nm and concentrations of ClO^- with excess TMB at pH 4.8 (acetic buffer).

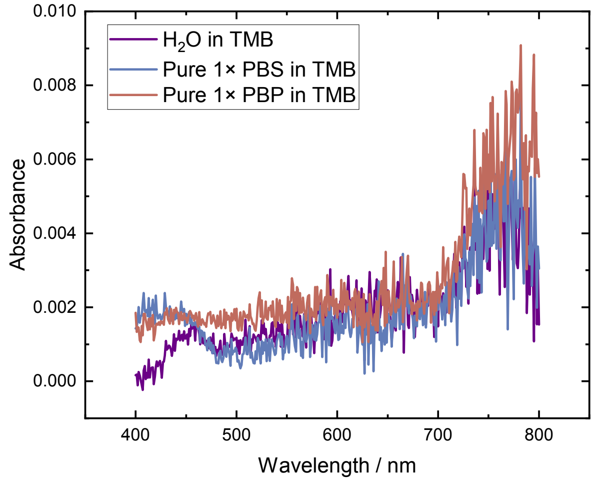

Figure S9. UV/Vis absorption spectra of excess TMB in pH 4.8 (acetic buffer) with water and pure salt solutions.

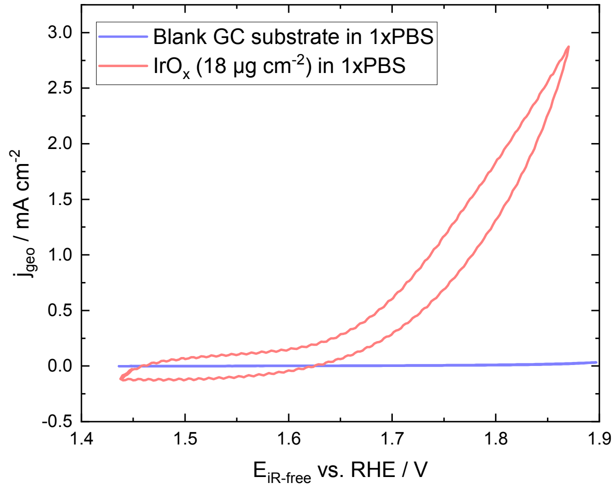

Figure S10. CV curves of blank glassy carbon substrate and IrO_x loaded on glassy carbon substrate in 1×PBS.

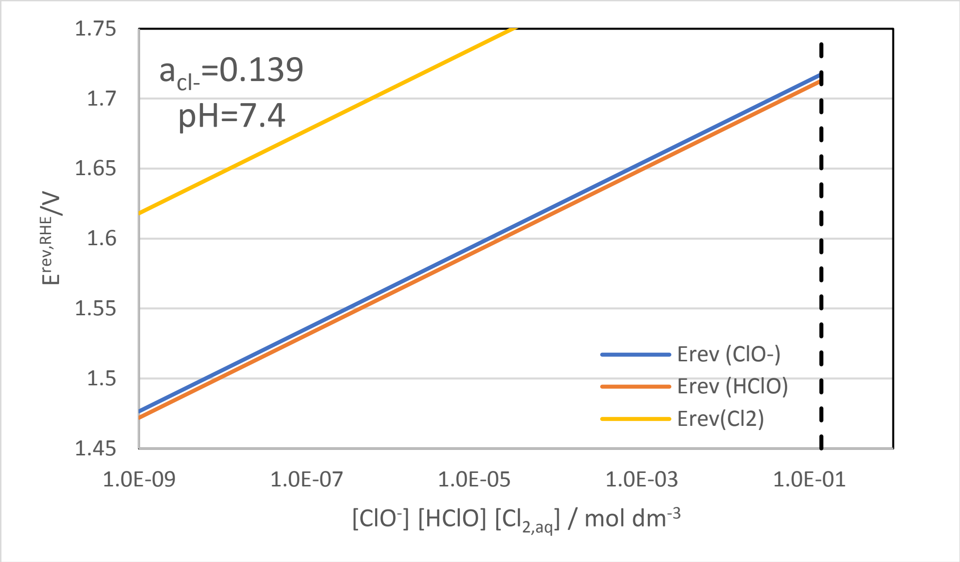

Figure S11. Reversible potentials (RHE scale) for hypochlorite formation at pH 7.4 under various hypochlorite concentrations, calculated using equations 1-3.

References

(1) Okada, T.; Abe, H.; Murakami, A.; Shimizu, T.; Fujii, K.; Wakabayashi, T.; Nakayama, M. A Bilayer Structure Composed of Mg|Co-MnO2 Deposited on a Co(OH)2 Film to Realize Selective Oxygen Evolution from Chloride-Containing Water. Langmuir 2020, 36 (19), 5227–5235. DOI: 10.1021/acs.langmuir.0c00547.

(2) Kong, L.-B.; Liu, M.-C.; Lang, J.-W.; Liu, M.; Luo, Y.-C.; Kang, L. Porous Cobalt Hydroxide Film Electrodeposited on Nickel Foam with Excellent Electrochemical Capacitive Behavior. J Solid State Electrochem 2011, 15 (3), 571–577. DOI: 10.1007/s10008-010-1125-6.

(3) McCrory, C. C. L.; Jung, S.; Peters, J. C.; Jaramillo, T. F. Benchmarking Heterogeneous Electrocatalysts for the Oxygen Evolution Reaction. J. Am. Chem. Soc. 2013, 135 (45), 16977–16987. DOI: 10.1021/ja407115p.

(4) Guo, Y.; Ma, Q.; Cao, F.; Zhao, Q.; Ji, X. Colorimetric Detection of Hypochlorite in Tap Water Based on the Oxidation of 3,3′,5,5′-Tetramethyl Benzidine. Anal. Methods 2015, 7 (10), 4055–4058. DOI: 10.1039/C5AY00735F.
